# Preventive Effect of *M. cochinchinensis* on Melanogenesis via Tyrosinase Activity Inhibition and p-PKC Signaling in Melan-A Cell

**DOI:** 10.3390/nu13113894

**Published:** 2021-10-29

**Authors:** Juyong Kim, Sung-Chul Hong, Eun Ha Lee, Jae Wook Lee, Seung-Hoon Yang, Jin-Chul Kim

**Affiliations:** 1 Natural Product Informatics Research Center, Korea Institute of Science and Technology (KIST), Gangneung 25451, Korea; juyongkim@kist.re.kr (J.K.); ehlee@kist.re.kr (E.H.L.); 2 Department of Agricultural Biotechnology, Seoul National University, Seoul 08826, Korea; 3 Smart Farm Research Center, Korea Institute of Science and Technology (KIST), Gangneung 25451, Korea; schong@kist.re.kr; 4 Natural Product Research Center, Korea Institute of Science and Technology (KIST), Gnagneung 25451, Korea; jwlee5@kist.re.kr; 5 Department of Medical Biotechnology, College of Life Science and Biotechnology, Dongguk University, Seoul 04620, Korea

**Keywords:** *M. cochinchinensis*, melanogenesis, tyrosinase, PKC signaling, melan-A

## Abstract

Whitening research is of particular interest in the cosmetics market. The main focus of whitening research is on melanogenesis inhibition through tyrosinase activity. The mechanism of melanogenesis is involved with tyrosinase activity and p-PKC signaling. In this study, we used *Momordica cochinchinensis* (*Lour.*) *spreng*, a tropical fruit found throughout Southeast Asia, to investigate the inhibitory effect of melanogenesis. *M. cochinchinensis* contains a high concentration of polyphenols, flavonoids, and unsaturated fatty acids, which might be related to antioxidant activity. This study aimed to determine whether *M. cochinchinensis* extracts inhibit melanin synthesis in melan-A cells by inhibiting tyrosinase activity and p-PKC signaling. *M. cochinchinensis* was divided into pulp and aril and extracted under various conditions, and it was confirmed that all pulp and aril extracts have high contents of both phenols and flavonoids. Melan-A cells were treated with PMA for three days to induce melanin synthesis. After PMA treatment, *M. cochinchinensis* extracts were added to cultured media in a dose-dependent manner. Melanin contents and MTS were used to determine the amount of melanin in live cells. *M. cochinchinensis* extracts were evaluated for their effects on tyrosinase activity and p-PKC signaling pathways by Western blotting. It was found that *M. cochinchinensis* extract treatment decreased the amount of melanin and suppressed p-PKC expression. Additionally, tyrosinase activity was reduced after *M. cochinchinensis* extract treatment in a dose-dependent manner. Therefore, it was concluded that *M. cochinchinensis* could be used in antimelanogenesis and functional cosmetic materials to improve whitening.

## 1. Introduction

Cosmetics aim to improve skin health, and recent trends in the cosmetics market show that there is a greater interest on whitening skin health [1]. Whitening is most closely related to melanin synthesis [2], which is a feedback response that protects from hazardous ultraviolet light [3]. However, reactive oxygen and nitrogen are produced during melanin synthesis, which can induce DNA lesions, cause skin cancer, and influence skin aging, such as wrinkles and freckles [4]. Melanin is produced from melanocytes, and in the melanosomes of melanocytes, tyrosinase converts tyrosine to dopaquinone, which becomes melanin. Thus, tyrosinase activity is a crucial target for preventing and treating melanin synthesis [5]. Many studies have demonstrated that tyrosinase activity inhibition is related to melanin synthesis. Additionally, the downstream signal transduction proteins of the p-PKC signaling pathway play an important role in melanin synthesis [6]. Melan-A has been used as a general in vitro model, and PMA stimulates melanogenesis and increases protein expression related to the p-PKC signaling pathway [7].

Phenolic chemicals, such as hydroquinone, are among the many substances that have excellent whitening properties [8,9]. However, various side effects of hydroquinone have been reported, including toxicity to normal skin fibroblasts and a dialectic effect on tissue after prolonged usage [10]. Therefore, its use as a primary chemical in whitening treatments is restricted and used exclusively in medicine. This has stimulated the search for natural products that overcome these disadvantages, have minor side effects on the human body, and have a significant whitening impact. The pear includes antioxidant-rich phenolic components such as arbutin, chlorogenic acid, and epicatechin, and is well-known for its outstanding whitening effects [11]. However, while cultivating a pear, the skin of a pear, which contains many phenolic compounds that have a whitening effect, is not recommend as an inner beauty material due to it having a large number of pesticide components and because of the problem of consumer recognition. Therefore, we chose the natural product for this study, which is easy to handle from an inner beauty standpoint and is frequently used in existing products.

The *M. cochinchinensis* is a species of the Cucurbitaceae family native to Southeast Asia and was discovered in Vietnam [12]. *M. cochinchinensis* produces red fruits in July and is harvested in August. *M. cochinchinensis* is known for its high beta-carotene and lycopene content. Its distinctive red color is widely used as a natural pigment in Vietnamese cooking and natural medicine [13]. Along with these carotenoid components, it contains various polyphenols, flavonoids, and fatty acids, with the aril portion being the most abundant in these compounds [14,15]. In addition to these components, *M. cochinchinensis* has functional effects, such as antioxidant, antimicrobial, anticancer, anti-inflammatory activities, and prevention of heart disease [16,17,18]. Despite exhibiting various valuable properties, no studies have reported the whitening impact of *M. cochinchinensis* in normal skin cell lines via the reduction of melanin synthesis and tyrosinase activity inhibition.

Thus, this study compared and evaluated the cytotoxicity of Melan-A and the ability to suppress melanin biosynthesis by *M. cochinchinensis*, which is known to have a high content of polyphenols and flavonoids. Additionally, *M. cochinchinensis* was evaluated as an inner beauty material based on the whitening effect by suppressing tyrosinase activity and modifying the p-PKC signaling pathway.

## 2. Materials and Methods

### 2.1. Preparation of M. cochinchinensis Extracts

#### 2.1.1. General Procedure

*M. cochinchinensis* fruits were collected from the northern Hong River area in Vietnam and were identified by Dr. Phuong Thien Thuong, Vietnam Korea Institute of Science and Technology (VKIST). The fruits were separated into skin, pulp, aril, and seed. The samples were air-dried at room temperature for several days and ground into fine particles using a blender. For accelerated solvent extraction (ASE), ASE 300 (Dionex, Sunnyvale, CA, USA) was used to extract *M. cochinchinensis* powder at 40 °C, with 15 min heating and 5 min preheating for three cycles.

#### 2.1.2. Preparation of Solvent Extract of Liquid Nitrogen-Treated Aril

The powdered arils of *M. cochinchinensis* were treated with 200 mL liquid nitrogen and stirred until liquid nitrogen was completely evaporated. After evaporation, the aril powders (5 g) were extracted with diethyl ether: CHCl_3_ = 1:2 (300 mL) in a 1-L beaker overnight. The suspension was filtered using Whatman filter paper grade 1 (GE healthcare, Chicago, IL, USA). The same procedure was repeated after adding diethyl ether and CHCl_3_. The suspension was combined and evaporated under reduced pressure to obtain an oily extract (332.9 mg, 6.65%).

#### 2.1.3. Preparation of ASE (S)

*M. cochinchinensis* (5 g) was treated with a cold isostatic press (500 MPa, 15 min). After pressure treatment, the pulp powder was extracted with 100% ethanol using ASE. The yellow solution was evaporated by reduced pressure to obtain an oily extract (51.9 mg, 1.04%).

#### 2.1.4. Preparation of ASE (L)

The pulp powder of *M. cochinchinensis* (5 g) was treated with a cold isostatic press (500 MPa, 15 min). After pressure treatment, the pulp powder was extracted with 100% ethanol using ASE. The solvent was evaporated to obtain to an oily extract (54.5 mg, 1.22%).

#### 2.1.5. Preparation of ASE-Nontreat

The pulp powder of *M. cochinchinensis* (5 g) was extracted with 100% ethanol using ASE system. The solvent was evaporated to obtain an oily extract (212.6 mg, 4.25%).

### 2.2. Total Polyphenol Content of M. cochinchinensis

The total polyphenol content was determined by spectrophotometry, using gallic acid as a standard. For this, 10 μL of either gallic acid or sample, 80 μL distilled water, 10 μL Folin–Ciocalteu reagent, and 100 μL Na_2_CO_3_ (70 g/L) were added to a 96-well microplate. These solutions were mixed on a shaker and incubated at 37 °C for 90 min. The absorbance was measured by a microplate spectrophotometer (Bio-Tek Power Wave XS, Winooski, VT, USA). The absorbance was measured at 750 nm. Results were expressed as milligrams of gallic acid equivalents per gram of total extract weight (mg GAE/g).

### 2.3. Total Flavonoid Content of M. cochinchinensis

The total flavonoid content was determined by spectrophotometry using catechin as a standard. First, 20 μL of either catechin or sample, 80 μL distilled water, and 6 μL NaNO_2_ (5%) were added to a 96-well microplate. Then, 6 μL AlCl_3_ (10%) was added after 5 min incubation at 37 °C. Furthermore, 40 μL NaOH (1 mol/L) and 48 μL distilled water were added after 6 min. These solutions were mixed on a shaker and incubated at 37 °C for 30 min. The absorbance was measured using a microplate spectrophotometer (Bio-Tek Power Wave XS, Winooski, VT, USA). The absorbance was measured at 510 nm. Results were expressed as milligrams of catechin equivalents per gram of total extract weight (mg CAE/g).

### 2.4. Melan-A Cell Culture

The Melan-A cell line was purchased from ATCC and was cultured in RPMI1640 media (Gibco, Carlsbad, CA, USA) containing 10% fetal bovine serum (HyClone Laboratories, Logan, UT, USA), 1% penicillin/streptomycin (HyClone Laboratories, Logan, UT, USA), and phorbol 12-myristate 13-acetate (PMA). To induce melanogenesis in melan-A cells, 3 × 10^4^ cells per well were cultured in six well plates, and 1 × 10^4^ cells per well were cultured in 96-well plates. After culturing for three days, *M. cochinchinensis* extract was applied to the cells for three days to measure melanin contents, 3-(4,5-dimethylthiazol-2-yl)-5-(3-carboxymethoxyphenyl)-2-(4-sulfophenyl)-2H-tetrazolium (MTS), and p-PKC signal protein levels.

### 2.5. Cell Viability

Melan-A, 1 × 10^4^ cells per well, were cultured in 96-well plates with PMA for three days; parts of the *M. cochinchinensis* extracts (15.625, 31.25, 62.5, 250, 500, and 1000 mg/mL) were applied to the cells for three days. MTS solution was added to the media and incubated for 2 h. Cytotoxicity was measured using a microplate spectrophotometer (Bio-Tek Power Wave XS, Winooski, VT, USA). The absorbance was measured at 490 nm.

### 2.6. Melanin Content Assay

Melan-A, 1 × 10^4^ cells per well, were cultured in 96-well plates with PMA for three days; parts of *M. cochinchinensis* (15.625, 31.25, 62.5, 250, 500, and 1000 mg/mL) were applied to the cells for three days and 0.5% trypsin with 0.1% triton X-100 was dissolved in the medium incubated for 30 min. Then, 50 mL 6 N NaOH was added to the cells and incubated for 30 min at 85 °C. The absorbance was measured at 490 nm using a microplate reader.

### 2.7. Tyrosinase Activity Assay

A substrate to determine tyrosinase inhibition activity was created. The reaction mixture contained 0.1 M sodium phosphate buffer, 1.5 mM L-tyrosine, and 1500 U/mL mushroom tyrosinase with sample (R) and without a sample (R’). The control mixture contained 0.1 M sodium phosphate buffer and 1.5 mM L-tyrosine with sample (C) and without a sample (C’). Mixtures were incubated for 15 min at 37 °C then put on ice to block the reaction. The absorbance was measured at 490 nm using a microplate reader. The tyrosinase inhibition activity was calculated using the following formula:

$$\%\;inhibition=100-\frac{Abs\;R-Abs\;C}{Abs\;R^{\prime }-Abs\;C^{\prime }}\times 100$$

### 2.8. Western Blotting

Melan-A, 3 × 10^4^ cells per well, were cultured in six-well plates with PMA for three days; parts of *M. cochinchinensis* (500 mg/mL) were applied to the cells for three days. The cells were harvested to evaluate the expression of p-proten kinase C (PKC), microphthalmia-associated transcriptian factor (MITF), tyrosinase-related protein 1 (TRP1), and tyrosinase-related protein 2 (TRP2). Cell lysates were prepared in radioimmunoprecipitation assay buffer (Thermo Scientific, Waltham, MA, USA). The protein concentrations were determined using the Bio-Rad protein assay (Bio-Rad, Hercules, CA, USA). Western blotting was conducted using 25 μg protein. Briefly, samples were separated using 12% sodium dodecyl sulfate-polyacrylamide gel electrophoresis and then transferred to a polyvinylidene fluoride membrane (Merck Millipore, Burlington, MA, USA; 0.4 μm). Membranes were blocked in 5% bovine serum albumin (Bovogen Biologicals, Keilor East, Australia) in tris-buffered saline and Tween-20 (Junsei Chemical, Tokyo, Japan) and incubated overnight at 4 °C with primary antibodies against p-PKC (Abcam; 1:1000), MITF (Santacruz; 1:1000), TRP1 (Santacruz; 1:1000), TRP2 (Santacruz; 1:1000), and glyceraldehyde 3-phosphate dehydrogenase (GAPDH, Cell signaling; 1:5000). After incubation with horseradish peroxidase-conjugated secondary antibodies (Abcam; 1:5000) for 1 h at room temperature, immunodetection was conducted using an enhanced chemiluminescence detection kit (GE Healthcare, Chicago, IL, USA).

### 2.9. Real-Time Polymerase Chain Reaction (RT-qPCR)

Total RNA was isolated from melan-A cells using an RNase mini kit (Qiagen, Valencia, CA, USA). Genomic DNA was removed by digestion with DNase I (Qiagen, Valencia, CA, USA). Reverse transcription was conducted using 1 μg mRNA per sample with the RevertAid First Strand cDNA Synthesis Kit (Thermo Fisher Scientific, Waltham, MA, USA). Gene expression was assessed with a SYBR Green PCR mixture with gene-specific oligonucleotide primers using an AB 7500 real-time PCR machine (Thermo Fisher Scientific, Waltham, MA, USA). Primer sequences and parameters are described in Table 1. The expression of the target genes was normalized to the expression of GAPDH, which was not significantly altered by treatments.

### 2.10. Statistical Analysis

Statistical analyses were conducted using SPSS v.19.0 for Windows (SPSS Inc., Chicago, IL, USA). Results were expressed as means ± standard error of the mean (SEM). Differences between means were analyzed by Student’s *t*-test.

## 3. Results

### 3.1. Total Phenol and Flavonoid Contents in Parts of M. cochinchinensis

We compared and analyzed the total polyphenol and flavonoid contents of *M. cochinchinensis* oil extract (Oil), aril-derived liquid nitrogen extract (LN2), and pulp-derived extract. As shown in Table 2, the total polyphenol content of an aril-derived extract is 1.16 mg GAE/g, while pulp-derived extract contains 4.97 mg GAE/g, 5.54 mg GAE/g, 3.04 mg GAE/g for ASE-nontreat (ASE), ASE-solid (ASE(S)), and ASE-liquid (ASE(L)), respectively. According to published research, the total polyphenol contents of the aril-, oil-, and pulp-derived extracts of *M. cochinchinensis* were 4.29 mg GAE/g, undetected, and 0.262 mg GAE/g, respectively [19]. When the aril-derived extract was compared with the existing report, it was confirmed that the concentration was lower, but the pulp-derived extract had a higher concentration. This phenomenon can be attributed to the influence of the *M. cochinchinensis*’s production area and the cultivation environment. However, when examining the same pulp-derived extract, the difference in the total polyphenol content depends on the extraction method. Therefore, the extraction method’s influence can determine the cause. As shown in Table 2, the total flavonoid content of the aril-derived extract was 16.31 mg CAE/g, that of the oil extract was 11.19 mg CAE/g, and the pulp-derived extract contained only 4.52 mg CAE/g for ASE-liquid.

### 3.2. M. cochinchinensis Suppresses Tyrosinase Activity

**Figure 1:**
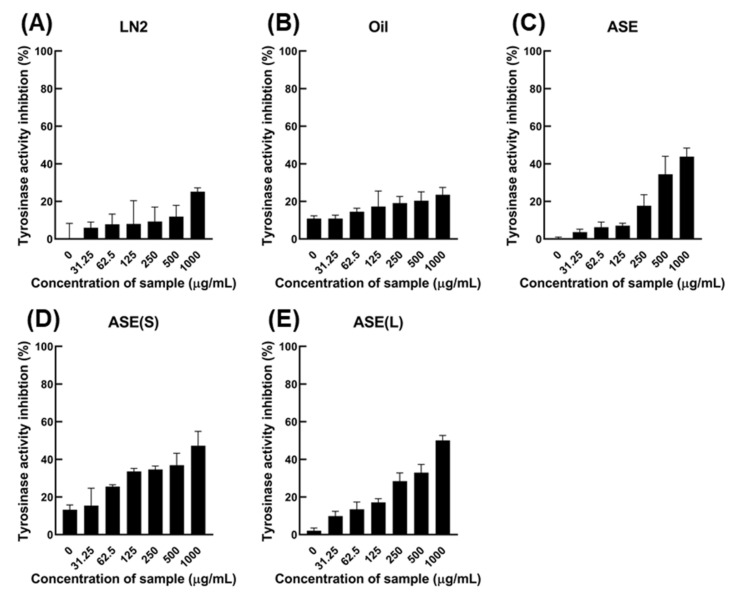
Evaluation of the effect of tyrosinase suppressing activity of *M. cochinchinensis* extracts. (**A**) Aril-derived LN2, (**B**) Seed-derived oil, (**C**) Pulp-derived accelerated solvent extract (ASE), (**D**) Pulp-derived ASE (S), and (**E**) Pulp-derived ASE (L).

The inhibitory effect of tyrosine activity was determined using L-tyrosine as a substrate and according to the extraction site and method of *M. cochinchinensis*. At a 500 μg/mL concentration, LN2, an aril site extract, effectively suppressed tyrosinase activities by 11.9%, and oil extracted from *M. cochinchinensis* was effective in suppressing tyrosinase activity by 20.4% (Figure 1). In the case of pulp-derived extracts, ASE-nontreat, ASE-solid, and ASE-liquid suppressed tyrosinase by 34.4%, 36.8%, and 32.9%, respectively. When the extract concentration was increased to 1000 μg/mL, the inhibitory effect on tyrosinase activity increased in proportion to the increase in LN2 and oil concentrations by 25.1% and 23.6%, respectively. The effect of ASE-nontreat, ASE-solid, and ASE-liquid on inhibiting tyrosinase activity increased by 43.8%, 47.3%, and 50.1%, respectively. Thus, tyrosinase activity inhibition showed a significant difference between pulp-derived and aril-derived extracts or oils, indicating a high level of tyrosinase activity inhibition. Alternatively, the difference in the inhibitory effect of tyrosinase activity was insignificant depending on the pulp extraction method.

### 3.3. M. cochinchinensis Reduces Melanin Content in Live Cells

**Figure 2:**
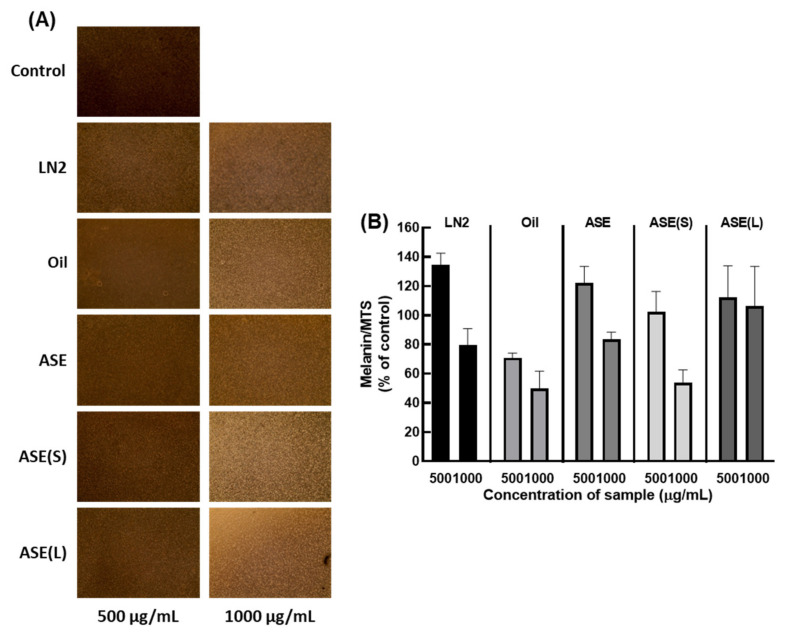
Effect of *M. cochinchinensis* on melanogenesis in melan-A cells. (**A**) Images of melan-A cells treated with parts of *M. cochinchinensis*. (**B**) Melanin contents per live cells. The results are presented as means ± SEM (*n* = 4 per group).

The contents of melanin and MTS were evaluated to determine the effect of *M. cochinchinensis* on melanogenesis in melan-A cells (Figure 2 and Figure S1). Except for ASE-liquid at 1000 μg/mL, the melanin content of live cells was significantly decreased (Figure 2). However, at 500 μg/mL, there were no significant differences in the melanin contents of live cells.

### 3.4. M. cochinchinensis Decreases Downstream Signal Transduction Protein Levels of the p-PKC Signaling Pathway

**Figure 3:**
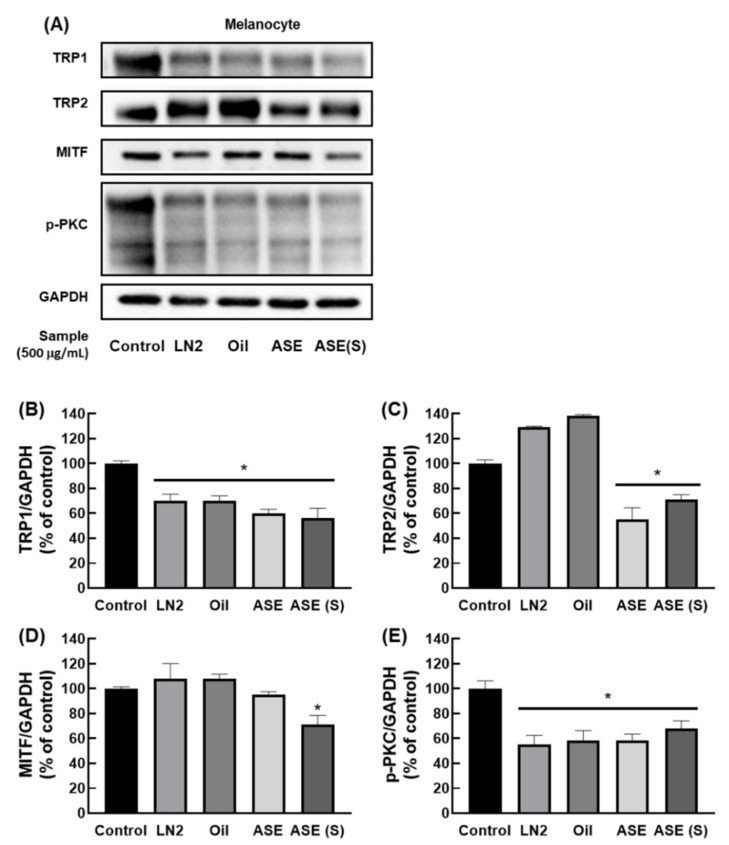
Effect of *M. cochinchinensis* on PMA-induced activation of the p-PKC signaling pathway in the melan-A cells. (**A**) Representative Western blots (*n* = 1 per lane) of p-PKC, MITF, TRP1, and TRP2 protein. (**B**–**E**) Western blot densitometry results for (**B**) TRP1, (**C**) TRP2, (**D**) MITF, and (**E**) p-PKC. The results are presented as means ± SEM (*n* = 3 per group). * *p* < 0.05 in compared to the control group.

**Figure 4:**
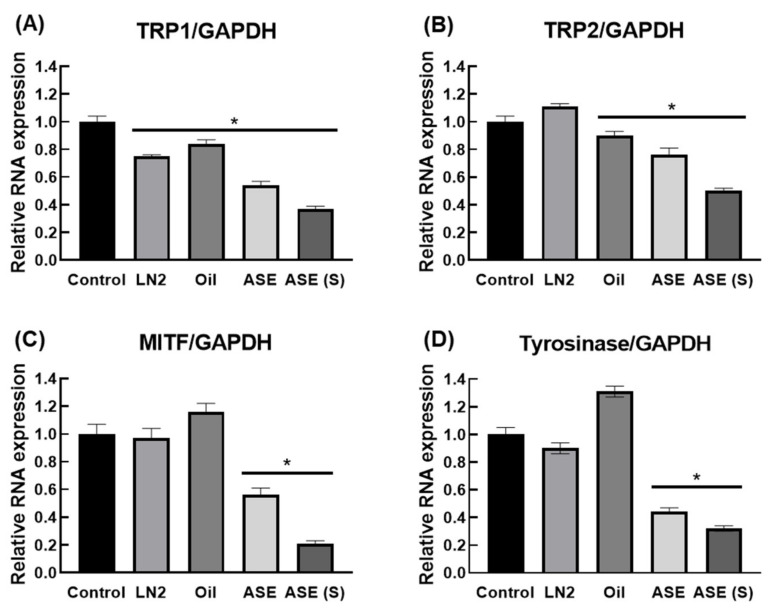
Effect of *M. cochinchinensis* on PMA-induced activation of the p-PKC signaling pathway in the melan-A cells. The RNA expression levels of (**A**) TRP1, (**B**) TRP2, (**C**) MITF, and (**D**) tyrosinase. The results are presented as means ± SEM (*n* = 3 per group). * *p* < 0.05 compared to the control group.

To determine whether *M. cochinchinensis* influences the p-PKC signaling pathway within melan-A, we examined the protein levels of p-PKC, MITF, TRP1, and TRP2. PMA-stimulated melan-A cells expressed increased p-PKC and TRP1, but *M. cochinchinensis* extract inhibited protein expression in all groups (Figure 3). However, TRP2 protein expression was inhibited only in pulp extracts, such as ASE-nontreat and ASE-solid, while MITF protein expression was significantly decreased in only ASE-solid. Additionally, the expression levels of tyrosinase, MITF, TRP1, and TRP2 were determined at the RNA expression level (Figure 4). Consequently, it was discovered that the level of TRP1 RNA expression decreased when *M. cochinchinensis* was treated. However, for TRP2, MITF, and tyrosinase, only ASE-nontreat and ASE-solid confirmed a significant decrease in RNA expression levels. These results indicate that the pulp site extract suppresses p-PKC signaling more than the aril and seed sites at the RNA and protein expression levels.

## 4. Discussion

We investigated the effects of *M. cochinchinensis* on melanogenesis using PMA. PMA was seen to induce the activation of PKC in melan-A cells, which aids in net formation and cell maturation [20]. When PKC is activated, the activity of tyrosinase increases, which increases melanin content [21]. However, inhibiting PKC activity simultaneously decreases tyrosinase activity and melanin content [22]. Because of this phenomenon, p-PKC signaling activates the DNA transcription factor, MITF, increasing the proteins TRP1, TRP2, tyrosinase, and the melanin proteins involved in melanin production [23]. Finally, p-PKC signaling promotes melanogenesis and results in blackening [24]. Consequently, we tried identifying the possibility of using *M. cochinchinensis* as an antimelanogenesis treatment material by inhibiting p-PKC signaling in this study.

Normally, natural products containing polyphenols and flavonoids have been reported [25]. Absorption of natural products containing many polyphenols and flavonoids can provide various health benefits, such as anticancer and antioxidant effects [26,27]. One of these favorable benefits is that it aids in manipulating defensive mechanisms by external elements, such as UV and infections [28]. The inhibition of PKC activity, a defensive mechanism, is another characteristic of natural compounds, including polyphenols and flavonoids [29]. Based on these features, there is a growing interest in natural products that are high in polyphenols and flavonoids in the whitening-related functional food and cosmetics markets; for example, daidzein, usually found in soybeans, is one such natural compound used in cosmetics. Additionally, it is used as a bleaching agent in PKC inhibitors [30,31].

This study confirmed that the aril, oil, and pulp of *M. cochinchinensis* contained many polyphenols and flavonoids, which were induced when the *M. cochinchinensis* extract was processed into PMA-induced melan-A cells. It was confirmed that p-PKC signaling associated with PKC was dramatically inhibited. Moreover, it was confirmed that the whitening mechanism of *M. cochinchinensis* extract dramatically decreased the expression levels of MITF, TRP1, TRP2, and tyrosinase, all of which are melanogenesis-regulating proteins. We also confirmed that melanogenesis was suppressed by treating melan-A cells induced by PMA, a PKC activator, with *M. cochinchinensis* because the polyphenols and flavonoids in *M. cochinchinensis* suppressed PKC. Therefore, this means that *M. cochinchinensis* has antimelanogenesis activity.

Thus, it was confirmed that *M. cochinchinensis* extracts inhibited PKC signaling, but tyrosinase inhibition activity was also assessed to determine whether it directly affects tyrosinase activities. According to previous reports [32,33], *M. cochinchinensis* extracts inhibited tyrosinase activity in enzymatic inhibition system, and this activity was confirmed in an in vitro study. As a results, this substantiated active inhibitory ability, as tyrosinase activity has a direct effect on melanin formation [34]. Melanin is created when tyrosine is metabolized into dopaquinone in the melanosome by tyrosinase and converted to melanin by TRP2 and TRP1 [35]. Additionally, it has been proven that, with melan-A cells, melanogenesis was inhibited by blocking tyrosinase. In summary, it has been demonstrated that *M. cochinchinensis* could directly inhibit tyrosinase activity and exert a whitening effect via PKC signaling suppression.

## 5. Conclusions

Natural products containing polyphenols and flavonoids are used as PKC inhibitors in whitening-related functional foods and cosmetics. A novel function for *M. cochinchinensis*, commonly consumed in Southeast Asia, has been found. It was discovered that the high concentration of polyphenols and flavonoids in *M. cochinchinensis* inhibits PKC, reduces the expression of melanogenesis-related proteins, and inhibits tyrosinase activity. Additionally, it has been confirmed that *M. cochinchinensis* reduces melanin production, the end-product of melanogenesis. Melan-A cells were treated with PMA with *M. cochinchinensis*, and the total melanin content in each living cell was determined. Consequently, it was established that both the aril and pulp-derived extracts of *M. cochinchinensis* contained significantly less melanin. Thus, *M. cochinchinensis* demonstrated a whitening effect by inhibiting melanin synthesis, suggesting that it may have potential as a functional food or cosmetic material derived from natural whitening agents.

## Figures and Tables

**Table 1:** List of primers.

Transcript	Forward/ Reverse	Primer Sequence	Annealing Temp. (°C)
MITF	F	5’-GGTGGATGGGATAAGGGAAAG-3’	54.9
	R	5’-AGGACCTTGAAAACCGACAG-3’	55.1
TRP-1	F	5’-GGTCTCCCTACATTTCCAGC-3’	55
	R	5’-AGCCCCAACTCTGTCTTTTC-3’	55.1
TRP-2	F	5’-GGAAGGAGTGAGCCAAGTTATG-3’	54.9
	R	5’-TCCAGAAGTTTGACAGCCC-3’	55.4
Tyrosinase	F	5’-GGGTTTTGGCTTTGTCATGG-3’	55.2
	R	5’-CTAACTTACTCAGCCCAGCATC-3’	54.9
GAPDH	F	5′-GCCAAGGTCATCCATGACAAC-3′	59.9
	R	5′-GTCCACCACCCTGTTGCTGTA-3′	56.4

**Table 2:** Total polyphenol and flavonoid contents of *M. cochinchinensis* extracts.

	LN2	Oil	ASE	ASE(S)	ASE(L)
Total polyphenol content (mg GAE/mL)	1.16 ± 1.06	-	4.97 ± 0.96	5.54 ± 0.97	3.04 ± 1.12
Total flavonoids content (mg catechin/mL)	16.31 ± 6.38	11.19 ± 4.42	-	-	4.52 ± 0.86

## Data Availability

Not applicable.

## Supplementary Materials

The following are available online at https://www.mdpi.com/article/10.3390/nu13113894/s1, Figure S1: Evaluation of melan-A cytotoxicity by extracts of *M. cochinchinensis*.
