# Supplementary figures and images for: Preventive Effect of M. cochinchinensis on Melanogenesis via Tyrosinase Activity Inhibition and p-PKC Signaling in Melan-A Cell
Source: Nutrients. 2021 Oct 29;13(11):3894. doi: 10.3390/nu13113894 (PMC8623224; doi:10.3390/nu13113894)

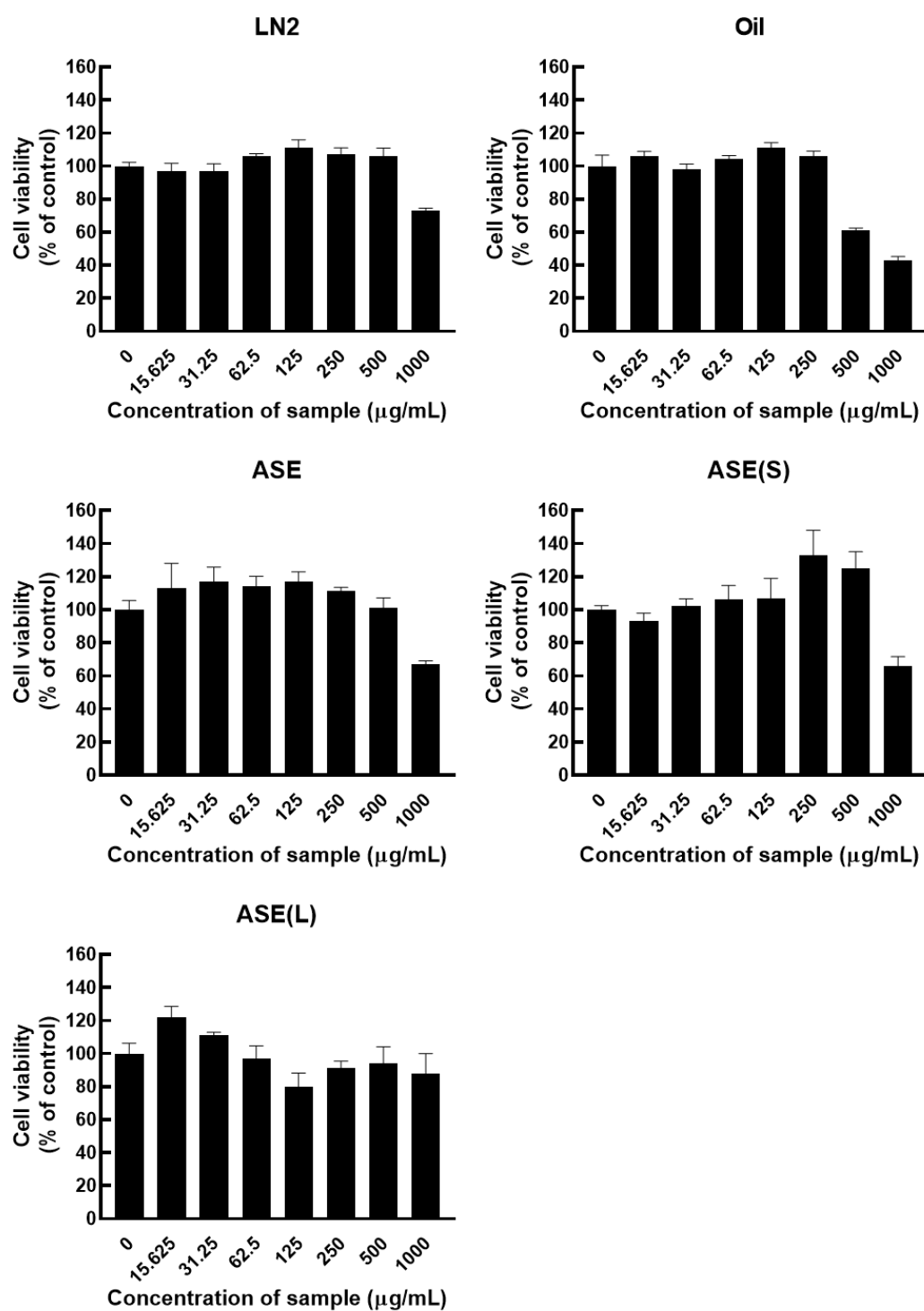

Figure S1. Evaluation of melan-A cytotoxicity by extracts of *M. cochinchinensis*.

Supplement: Supplementary file 1 [file nutrients-13-03894-s001.zip › nutrients-1425739-supplementary.pdf]
